# Protective effects of Atractylodes macrocephala polysaccharides on acetaminophen-induced liver injury

**DOI:** 10.3389/fphar.2025.1583334

**Published:** 2025-06-19

**Authors:** Jiali Wu, Biao Jia, Shuai Gong, Yangpeng Li, Jiaqi Wang, Yuqiao Huang, Jiao Guo

**Affiliations:** Guangdong Metabolic Diseases Research Center of Integrated Chinese and Western Medicine, Key Laboratory of Glucolipid Metabolic Disorder, Ministry of Education, Guangdong TCM Key Laboratory for Metabolic Diseases, Guangzhou Higher Education Mega Center, Institute of Chinese Medicine, Guangdong Pharmaceutical University, Guangzhou, China

**Keywords:** hepatoprotection, herbal polysaccharides, intestinal microbiota, oxidative stress, NLRP3 inflammasome

## Abstract

**Background:**

Drug-induced liver injury (DILI) is a major clinical concern due to its unpredictable nature and lack of effective therapeutic options.

**Methods:**

This study investigated the hepatoprotective effects of Atractylodes macrocephala polysaccharides (AMPs) in a mouse model of acetaminophen (APAP)-induced liver injury. Mice were pretreated with AMPs for 7 days prior to APAP challenge, and liver injury was evaluated through histopathology, serum biochemistry, molecular assays, and gut microbiota analysis.

**Results:**

AMPs treatment significantly reduced serum alanine aminotransferase (ALT) and aspartate aminotransferase (AST) levels compared to the APAP group (*p* < 0.05). Hepatic oxidative stress was alleviated, as indicated by increased levels of glutathione (GSH, *p* < 0.05) and superoxide dismutase (SOD, *p* < 0.05), and reduced malondialdehyde (MDA, *p* < 0.05). AMPs also suppressed inflammatory cytokines, including *Il-1β*, *Tnf-α*, *Il-6*, and *Nlrp3* (*p* < 0.05), and modulated apoptosis-related proteins by downregulating *Bax* and upregulating *Bcl-2* and *Bcl-xl* expression (*p* < 0.05). Furthermore, AMPs improved gut microbiota diversity and enriched beneficial genera such as *Roseburia*, as revealed by 16S rDNA sequencing. Fecal microbiota transplantation from AMPs-treated mice replicated these hepatoprotective effects, highlighting the involvement of the gut–liver axis.

**Conclusion:**

These findings support the therapeutic potential of AMPs as a multifaceted agent for DILI, exerting protective effects through modulation of oxidative stress, inflammation, apoptosis, and intestinal dysbiosis.

## 1 Introduction

Drug-induced liver injury (DILI) represents a broad range of liver damage caused by various agents including prescription drugs, over-the-counter medications, and supplements. Clinical manifestations range from mild, often asymptomatic liver enzyme elevations to severe acute liver failure, potentially fatal (Andrade et al., 2019). With increasing drug availability and self-medication trends, DILI incidents are rising, involving over 30,000 drug types with more than 3% posing liver injury risks (Avigan, 2014). DILI poses significant public health challenges, complicated by its nonspecific symptoms and the absence of reliable biomarkers, making early detection and effective treatment challenging, often resulting in treatment delays (Andrade et al., 2019; Shen et al., 2019).

Acetaminophen (APAP), known as paracetamol, has been a common antipyretic and analgesic globally since 1884 (Brune, Renner and Tiegs, 2015). While safe at recommended doses, overdoses can lead to significant liver toxicity and are a major cause of acute liver failure in developed countries, accounting for 45.7% of cases in North America and 65% in the UK. N-acetyl-L-cysteine (NAC), a glutathione precursor, is a US FDA-approved first-line treatment for APAP overdose (Budnitz, Lovegrove and Crosby, 2011; Manthripragada, Zhou, Budnitz, Lovegrove and Willy, 2011; Stravitz and Lee, 2019). Its effectiveness is highly time-sensitive, with the best outcomes when administered within 8 h post-overdose. Delays can diminish NAC’s efficacy, potentially leading to liver failure or necessitating liver transplantation (Björnsson, 2021; Du, Ramachandran and Jaeschke, 2016; Rushworth and Megson, 2014). Ongoing research into APAP’s toxic mechanisms is essential to developing targeted treatments for acetaminophen-induced liver injury (AILI), aiming to improve therapeutic outcomes significantly.

Polysaccharides, widely distributed in the roots, stems, leaves, flowers, fruits, and seeds of plants, exhibit numerous biological activities, including antioxidant, anti-tumor, immune modulation, anti-aging, anti-allergic, and antiviral effects. Many polysaccharides are low in toxicity and highly safe, making them valuable in clinical applications (Liu et al., 2015; Sun et al., 2015). Notably, plant polysaccharides have shown protective effects against liver injury (Liu et al., 2019; Sun et al., 2015; Wu, Fan, Huang, Wu and Guo, 2018; Zhao et al., 2019). Atractylodes macrocephala polysaccharides (AMPs) have demonstrated a broad range of biological functions, including anti-inflammatory, antioxidant, hepatoprotective, anti-allergic, antiviral, immune-regulatory, antithrombotic, anti-obesity, antimicrobial, and anticancer properties (Deng, Zheng, Yun, Zhang and Yang, 2019; Kim et al., 2011; Song et al., 2014; Zheng et al., 2018). Several studies suggest AMPs’ protective effects against liver damage caused by various factors. Guo et al. found that AMPs alleviate LPS-induced liver injury in mice by activating the TLR4-MyD88-NF-κB pathway, reducing inflammation and oxidative stress (Guo et al., 2021). Han et al. also reported AMPs’ protective effects in chemical liver injury models, such as those induced by carbon tetrachloride (Han et al., 2016). These findings support the rationale for evaluating AMPs in the context of APAP-induced liver injury, providing a mechanistic basis for subsequent investigation into their therapeutic potential. Thus, this study aims to clarify the role of mitigating APAP-induced acute liver injury and to explore the underlying mechanisms involved.

Therefore, the present study aims to systematically investigate the protective effects of AMPs against AILI in mice, with a particular focus on elucidating the mechanistic contributions of anti-oxidative, anti-inflammatory, anti-apoptotic, and gut microbiota-modulatory pathways. By integrating biochemical, histopathological, molecular, and microbiological approaches, we seek to establish AMPs as a promising multi-targeted therapeutic agent for drug-induced liver injury.

## 2 Materials and methods

### 2.1 Materials and reagents

Atractylodes macrocephala Koids were sourced from Kangmei Pharmaceutical Co., Ltd. APAP (A7250-10G) and N-acetylcysteine (NAC) (L2007325) were procured from Sigma, St. Louis, MO, United States. Nuclease-free water (BL510A) and HE dye solution (BL700B) were obtained from Biosharp, Beijing, China. The PrimeScript™ RT Reagent Kit (RR037A) was acquired from TAKARA, Japan, and the Soil DNA Kit (D6922-00) was secured from Omega Bio-Tek, Norcross, Georgia, United States. The TruSeq DNA SBZ-Wle Prep Kit (FC-121) was sourced from ILLUMINA, San Diego, CA, United States, and SYBR Green Master MIX (1725125) was purchased from Bio-Rad, Hercules, CA, United States. The BCA protein quantification kit (23,225) was sourced from Thermo Fisher Scientific (Massachusetts, United States).

Antibodies such as Anti-IL-1β (bs-0812R) were sourced from Bioss, Beijing, China. Anti-Bcl-2 (BA0412) and Anti-CD68 (BA3638) were purchased from Boster, Wuhan, China. Anti-Bax (60267-1-Ig) was purchased from Proteintech, Wuhan, China. Anti-mouse Anti-Rabbit IgG (H + L) HRP Conjugate (W4021) and Anti-rabbit Anti-Rabbit IgG (H + L) HRP Conjugate (W4011) were procured from Promega, Madison, WI, United States. Anti-β-actin (HC201-02) was obtained from TransGen, Beijing, China.

### 2.2 Monosaccharide composition analysis

Ion chromatography (ICS5000, Thermo Fisher Scientific, United States) was utilized to analyze the monosaccharide composition of AMPs. A 6 mg of AMPs was subjected to hydrolysis at 121°C with 2 M trifluoroacetic acid for 2 h. Nitrogen gas was introduced to the mixture, which was then evaporated. The samples was cleaned by adding 99.99% methanol and repeating the procedure three times before drying. For testing, the sample was dissolved in sterile water to minimize contamination and subsequently transferred into a chromatographic vial. The chromatographic column used was the Dionex™ CarboPac™ PA20 (150 × 3.0 mm, 10 μm). The injection volume was 5 μL. Mobile phase A was H_2_O; mobile phase B was 0.1 M NaOH; and mobile phase C was 0.1 M NaOH with 0.2 M sodium acetate (NaAc). The flow rate was set at 0.5 mL/min. The mobile phase gradient followed this schedule: 26 min, A/B/C (85:5:10, V/V); 42 min, A/B/C (85:5:10, V/V); 42.1 min, A/B/C (60:0:40, V/V); 52 min, A/B/C (60:40:0, V/V); 52.1 min, A/B/C (95:5:0, V/V); and 60 min, A/B/C (95:5:0, V/V), with an elution flow rate of 0.5 mL/min and a column temperature of 30°C.

### 2.3 Fourier transform infrared spectroscopy (FT-IR)

FT-IR spectroscopy was used to analyze the functional groups and structural characteristics of AMPs. Briefly, an appropriate amount of freeze-dried AMPs powder was thoroughly mixed with dried potassium bromide (KBr) in a ratio of 1:100 (w/w), ground evenly, and pressed into a transparent pellet using a tableting machine. The pellet was then scanned using an FT-IR spectrometer (Bruker, Germany) in the range of 4,000–400 cm^-1^. This technique was employed to identify characteristic absorption peaks corresponding to functional groups such as hydroxyl (O–H), carboxyl (C=O), and glycosidic (C–O–C) linkages, which are indicative of polysaccharide structures. FT-IR analysis provided supportive structural evidence of AMPs and facilitated the confirmation of its polysaccharide composition.

### 2.4 Scanning electron microscopy

AMPs samples were sputter-coated with gold under vacuum and examined using a scanning electron microscope (Zeiss Merlin Compact, Jena, Germany) at magnifications ranging from 100 to 4,000 times, with an acceleration voltage of 5.0 kV.

### 2.5 Animal experiments

A total of 30 male Balb/c mice, aged 6–8 weeks, were purchased from Beijing Weitahe Experimental Animal Technology Co., Ltd. For the fecal microbiota transplantation (FMT) experiments, additional male Balb/c mice, aged 6–8 weeks, were obtained from Guangdong Experimental Animal Center. All mice were housed in the Animal Experiment Center at Guangdong Pharmaceutical University under specific pathogen-free (SPF) conditions. The mice were provided unrestricted access to water and standard rodent food. The feeding environment was maintained at a temperature of 25°C–26°C and a humidity level of 55%–65%. All experimental procedures involving animals were conducted in strict accordance with Chinese animal welfare regulations and were approved by the Animal Ethics Committee of Guangdong Pharmaceutical University (experimental ethics review number: gdpulacspf 2022021).

After 1 week of adaptive feeding, mice were randomly assigned to 5 groups: the normal control group (CON, n = 6), the APAP 12-h model group (n = 6), the positive control drug NAC 12-h model group (n = 6), the low-concentration AMPs group (50 mg/kg, n = 6), and the high-concentration AMPs group (100 mg/kg, n = 6) (Qian, Jiang, Luo and Jiang, 2023). Mice in the AMPs treatment groups were pretreated with AMPs and NAC via gavage for 7 days prior to APAP modeling. APAP was freshly prepared on the day of administration by dissolving the powder in sterile normal saline (0.9% NaCl) to a final concentration of 15 mg/mL. The solution was administered intraperitoneally at a dose of 250 mg/kg based on body weight. All APAP solutions were prepared within 30 min before injection to ensure stability. Both NAC and AMPs were dissolved in sterile normal saline prior to oral gavage. The solutions were freshly prepared daily, and complete dissolution was confirmed before administration. In contrast, mice in the other groups received equal volumes of physiological saline using the same method. On the final day of pretreatment, all mice were fasted for 22 h with access to water only. Following the fasting period, they were allowed to resume a normal diet. Except for the CON group, all other groups received an intraperitoneal injection of APAP solution (250 mg/kg) based on their body weight, while the CON group received an equivalent volume of physiological saline. Twelve hours after APAP model induction, blood and liver were collected, immediately frozen in liquid nitrogen, and stored at −80°C for further analysis.

In the fecal microbiota transplantation (FMT) experiment, 14 mice were randomly divided into two groups: the FMT^WT^ (WT) group (n = 5) receiving fecal microbiota from normal mice, and the FMT^AMPs^ (AMPs) group (n = 5) receiving microbiota from AMPs-treated mice. Five days prior to the experiment, all mice underwent a pre-treatment regimen of antibiotics (vancomycin 100 mg/kg, neomycin 200 mg/kg, metronidazole 200 mg/kg, and ampicillin 200 mg/kg) via gavage (Sim et al., 2022). On the final day of AMPs pre-treatment, feces were collected from the AMPs and CON group mice to serve as donors. Approximately 200 mg of fresh feces was mixed with sterile saline, centrifuged, and the supernatant was administered orally via gavage for three consecutive days. On the final gavage day, mice were fasted for 22 h but permitted to drink water. After fasting, they resumed their diet and received an intraperitoneal injection of APAP solution (250 mg/kg), with a dosing volume of 20 μL per gram of body weight. At the end of the experimental period, mice were euthanized under anesthesia, and blood and liver tissues were collected for biochemical, histopathological, and molecular analyses.

### 2.6 Biochemical detection of serum and tissue samples

Blood samples were centrifuged at 3,000 rpm for 10 min using a centrifuge (TDZ5-WS, Xianyi, Hunan, China) to separate the serum, which was collected from the supernatant. Liver tissues were processed using an automatic glass grinder to produce homogenates, which were subsequently centrifuged at 2,000–2,500 rpm for 15 min to extract the supernatant. The concentrations of alanine aminotransferase (ALT) and aspartate aminotransferase (AST) in the mouse serum were determined using specific assay kits (C009-2-1 for ALT and C010-2-1 for AST, both from Nanjing, China). Additionally, the levels of malondialdehyde (MDA, A003-1-2), superoxide dismutase (SOD, A001-3-2), and glutathione (GSH, A006-2-1) were measured using commercial colorimetric kits (Nanjing Jiancheng Bioengineering Institute, Nanjing, China) according to the manufacturer’s instructions, to assess oxidative stress.

### 2.7 Histological examinations

Liver tissues collected for analysis were first fixed in a fixative solution for 24 h, followed by dehydration and embedding in paraffin. The tissues were then sectioned into 4 µm slices. Hematoxylin and eosin (H&E) staining was employed to visualize the tissue structure. The stained sections were examined under an Olympus BX-51 microscope (Olympus, Tokyo, Japan).

### 2.8 Immunohistochemistry

Liver sections were processed with a 15-min high-temperature and high-pressure antigen retrieval in citrate buffer, followed by a 10-min block with 3% H_2_O_2_ at room temperature. The sections were then treated with 1% BSA for 1 h at 37°C, incubated overnight with CD68 primary antibody at 4°C, followed by a 1-h incubation with horseradish peroxidase-labeled secondary antibody at room temperature. The sections were developed with DAB for 30 s to 2 min, counterstained with hematoxylin, dehydrated, and mounted. Observations and photographs were taken under 10X and 20X objectives using an Olympus microscope.

### 2.9 TUNEL staining

A similar procedure was followed using the TUNEL immunohistochemistry kit (KGA702, Nanjing, China). After initial preparation, slices were incubated with Stretavidin HRP for 30 min at 37°C in the dark, developed with DAB, counterstained with hematoxylin, dehydrated, and sealed on slides. Images were captured under the Olympus microscope.

### 2.10 Quantitative real-time PCR analysis (q-PCR)

Total RNA was extracted from liver tissue using TRIzol reagent (Invitrogen, MA, Waltham, United States) and reverse transcription was performed. cDNA synthesis was carried out using the cDNA Eraser assay kit (Takara, Beijing, China) with the extracted mRNA as the template. PCR amplification products were quantified using SYBR Green Supermix (EZBioscience, Roseville, United States). qPCR detection was performed following the standard protocol on a real-time PCR system (LightCycler 480 Instrument II, Roche Diagnostics, Basel, Switzerland). β-actin was used as the internal reference for normalization. The specific primer sequences used in the analysis are listed in Supplementary Table S1.

### 2.11 Western blot analysis

Total protein was extracted from liver tissues using RIPA lysis buffer containing protease inhibitors, and protein concentrations were determined using a BCA protein assay kit. Equal amounts of protein (50 μg per lane) were separated by SDS-PAGE and transferred onto PVDF membranes. After blocking with 5% non-fat milk for 1 h at room temperature, the membranes were incubated overnight at 4°C with primary antibodies diluted at 1:1,000. After washing, membranes were incubated with HRP-conjugated secondary antibodies diluted at 1:3,000 for 1 h at room temperature. Protein bands were visualized using enhanced chemiluminescence (ECL) reagents, and images were captured using a chemiluminescence imaging system. Band intensity was analyzed using ImageJ software, and target protein expression levels were normalized to β-actin.

### 2.12 16S rDNA sequencing

After extracting DNA from the fecal microbiome using the Metabo-Profile kit, PCR amplification products were purified using Vazyme VAHTSTM DNA Clean Beads. The sequencing library was then prepared using Illumina’s TruSeq Nano DNA LT library preparation kit. Finally, sequence denoising or OTU clustering was performed using the QIIME2 data analysis process or the Vsearch software analysis process.

### 2.13 Statistical analysis

All data are presented as mean ± standard deviation (mean ± SD), and statistical analysis was performed using GraphPad Prism 9.0 software. Differences between two groups were analyzed using the t-test, while differences among multiple groups were assessed using one-way ANOVA. A *p* < 0.05 was considered statistically significant.

## 3 Results

### 3.1 Chemical characteristics of AMPs

**FIGURE 1:**
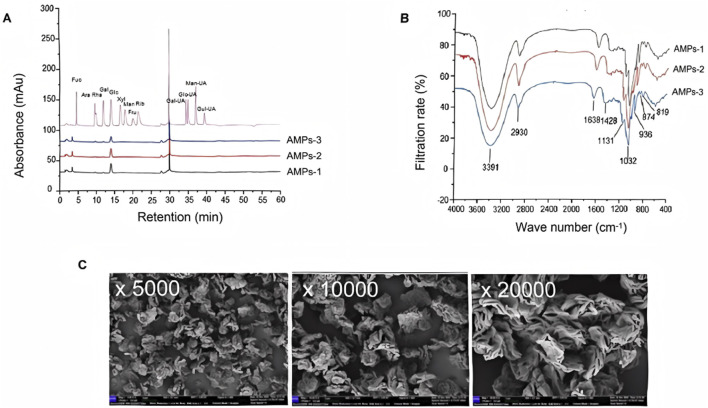
Characterization of AMPs. **(A)** Ion chromatogram of the monosaccharide composition of AMPs. Ara, Arabic sugar; Fuc, Fucoidan; Gal, Galactose; Gal-UA, Galacturonic acid; Glc, glucose; Glc-UA, Glucuronic acid; Man, Mannose; Xyl, Xylose. **(B)** Fourier-infrared spectra of AMPs. **(C)** SEM images of AMPs.

In terms of the monosaccharide composition, the primary component is glucan, with glucose (Glc) comprising 83.92% of the molecular weight. Other monosaccharides present include arabinose (Ara), galactose (Gal), galacturonic acid (Gal UA), and glucuronic acid (Glc UA), which account for 7.19%, 5.47%, 3.00%, and 0.41%, respectively (Figure 1A). These findings indicate that AMPs possess a relatively pure structure, making them suitable for further analysis of their glycosidic bond types via methylation.

The FTIR results, recorded in the frequency range of 400–4,000 cm^-1^, reveal several characteristic features (Figure 1B). The vibration region between 3,200–3,600 cm^-1^ corresponds to the hydroxyl group (-OH) stretch. A fluctuation in the absorption peak at 2,930 cm^-1^ is observed, associated with the carbon-hydrogen (C-H) bond, alongside stretching vibrations of CH, CH_2_, and CH_3_ within the 2,800–3,000 cm^-1^ range. Specifically, vibrations in the 905–876 cm^-1^ range indicate the presence of beta-configured beta-glucans, while those in the 855–833 cm^-1^ range suggest the presence of alpha-configured glucans. Additionally, vibrations between 1,000–1,200 cm^-1^ may arise from the ester sugar group (C-O-C) and the C-O-H bond in the pyran ring, confirming that AMPs contain a pyran ring structure. In addition, at magnifications of×5,000, ×10,000, and ×20,000, AMPs are observed under electron microscopy as irregularly distributed, exhibiting multi-layered or rose petal-like shapes (Figure 1C). The surface appears rough and loose.

### 3.2 AMPs treatment alleviates APAP-induced liver injury and hepatocyte apoptosis

**FIGURE 2:**
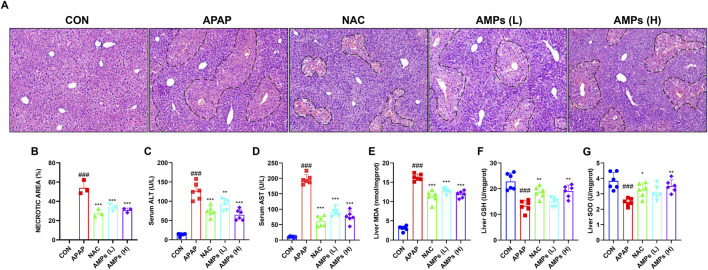
AMPs improves the histopathological and biochemical indicators of APAP-induced liver injury in mice. **(A)** HE stained section (×10 magnification). **(B–G)** Statistics of the area of pathological necrosis area of the model liver, serum ALT and AST levels, and liver GSH/SOD/MDA levels at 12 h. Data of biochemical indexes were presented as mean ± SD (n = 6). # represents compared with CON group; ###*p* < 0.001; * indicates compared with APAP group; *p < 0.05, **p < 0.01, ***p < 0.001.

Histological analysis using hematoxylin and eosin (H&E) staining showed that APAP administration caused extensive hepatocellular necrosis and structural disruption. In contrast, both NAC and AMPs treatments notably alleviated these pathological changes, with improved hepatic architecture and reduced necrotic areas, particularly in the high-dose AMPs group (Figures 2A,B).

Serum biochemical indicators further confirmed these histological findings. ALT and AST levels were markedly elevated following APAP exposure, indicating severe hepatic injury. Treatment with AMPs significantly lowered the concentrations of these enzymes, demonstrating a protective effect on liver function (Figures 2C,D).

In terms of oxidative stress, APAP induced a pronounced decrease in GSH and SOD levels and a concomitant increase in MDA, reflecting enhanced lipid peroxidation. Administration of AMPs restored the antioxidant capacity of the liver, as evidenced by increased GSH and SOD levels and decreased MDA concentrations (Figures 2E–G). These results suggest that AMPs mitigate APAP-induced liver injury by reducing oxidative stress and preserving hepatocellular integrity.

**FIGURE 3:**
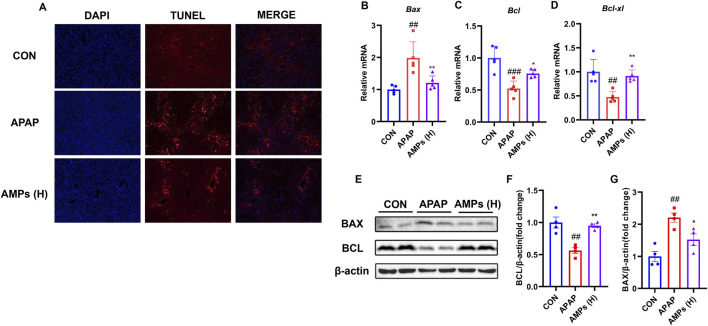
AMPs improves APAP-induced apoptosis of mouse hepatocytes. **(A)** TUNEL staining results of paraffin sections of mouse liver tissue. **(B–D)** The *Bax* mRNA, *Bcl-xl* mRNA and *Bcl* mRNA expression levels in mouse liver tissue (n = 5). **(E)** Protein expression levels of BCL and BAX in mouse liver tissue. **(F)** Fold change of Bcl protein. **(G)** Fold change of Bax protein. Data of biochemical indexes were presented as mean ± SD (n = 6). #represents compared with CON group; ##*p* < 0.01, ###*p* < 0.001; *indicates compared with APAP group; *p < 0.05, **p < 0.01.

To further investigate the protective effect of AMPs on APAP-induced hepatocyte apoptosis, we performed TUNEL staining on liver tissue and evaluated the mRNA and protein levels of apoptosis markers BAX and BCL-2. The results showed that compared with the control group, APAP significantly induced apoptosis in mouse liver cells. However, AMPs treatments effectively alleviated this effect when compared to the APAP group (Figure 3A).

Additionally, APAP was found to significantly upregulate the pro-apoptotic factor BAX in the liver, while downregulating the mRNA and protein levels of the anti-apoptotic factor BCL-2. AMPs treatment, on the other hand, significantly reduced the mRNA and protein levels of BAX and increased the expression of BCL-2, thereby alleviating liver cell apoptosis (Figures 3B–G). These findings suggest that AMPs have a protective effect against APAP-induced hepatocyte apoptosis by modulating the expression of key apoptosis markers, BAX and BCL-2, and may serve as a potential therapeutic agent in alleviating liver injury.

### 3.3 AMPs inhibits APAP-induced liver inflammation in mice

**FIGURE 4:**
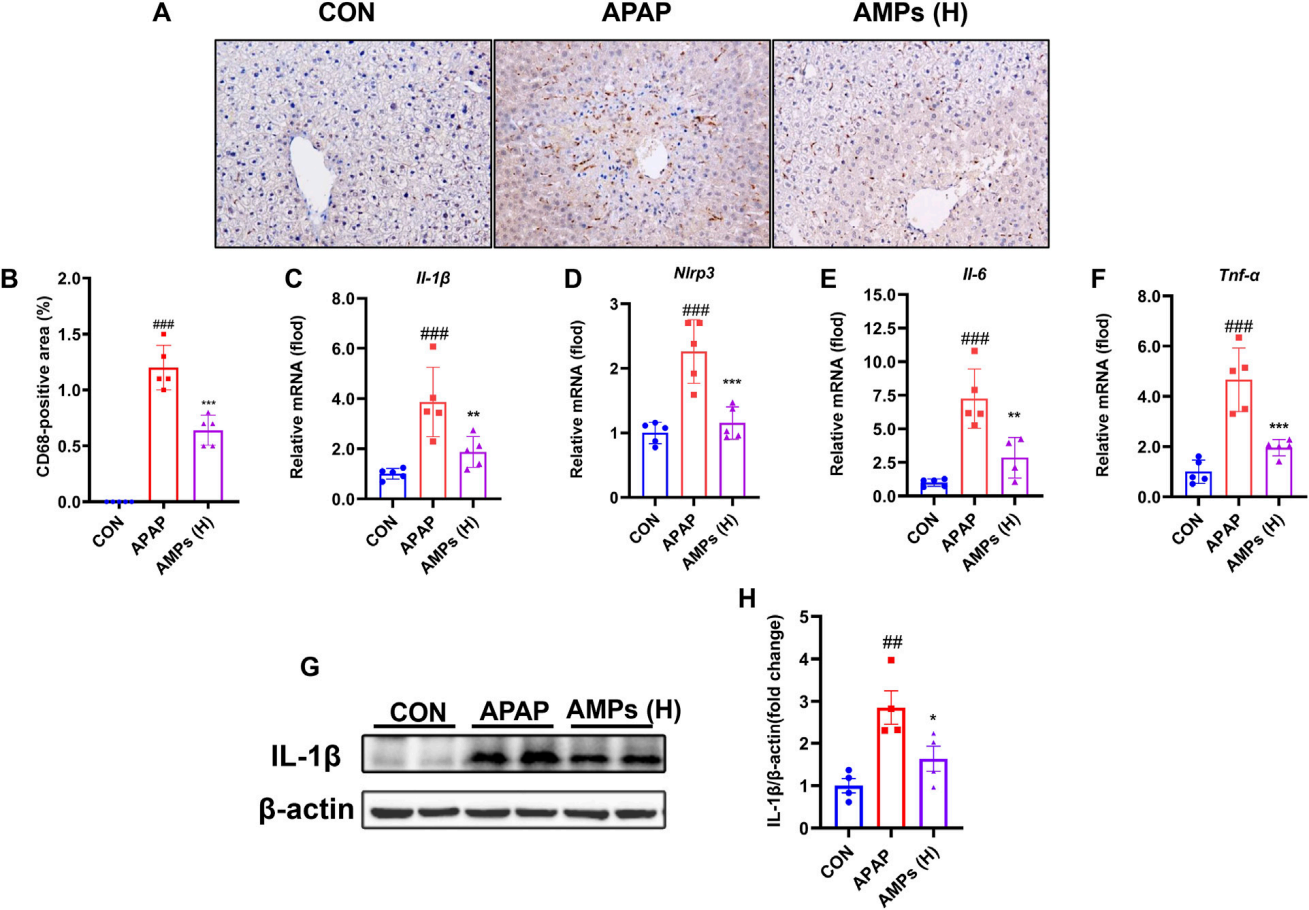
AMPs improves APAP-induced liver inflammation in mice. **(A)** Immunohistochemical staining results of CD68 in mouse liver tissue. **(B)** Statistical results of CD68-positive area in mouse liver tissue. **(C–F)** The *IL-1β* mRNA, *Nlrp3* mRNA, *IL-6* mRNA and *TNF-α* mRNA expression levels in mouse liver tissue (n = 5). **(G)** Protein expression level of IL-1β in mouse liver tissue. **(H)** Fold change of IL-1β protein. Data of biochemical indexes were presented as mean ± SD (n = 5). # represents compared with CON group; ##*p* < 0.01, ###*p* < 0.001; * indicates compared with APAP group; *p < 0.05, **p < 0.01, ***p < 0.001.

To evaluate the anti-inflammatory potential of AMPs, hepatic macrophage infiltration and inflammatory cytokine expression were assessed. Immunohistochemical staining revealed a substantial accumulation of CD68-positive macrophages in the livers of APAP-treated mice. This response was markedly attenuated by AMPs administration, indicating a reduction in inflammatory cell infiltration (Figures 4A,B).

At the molecular level, APAP treatment led to a significant upregulation of inflammatory mediators, including *Il-1β*, *Il-6*, *Tnf-α*, and *Nlrp3*. AMPs treatment effectively suppressed the transcription of these cytokines (Figures 4C–F). Western blot analysis further confirmed that the elevated IL-1β protein expression induced by APAP was attenuated following AMPs intervention (Figures 4G,H). These findings indicate that AMPs exert anti-inflammatory effects in the context of APAP-induced liver injury by suppressing both immune cell infiltration and pro-inflammatory signaling pathways.

### 3.4 AMPs restores AILI induced gut microbiota disruption

**FIGURE 5:**
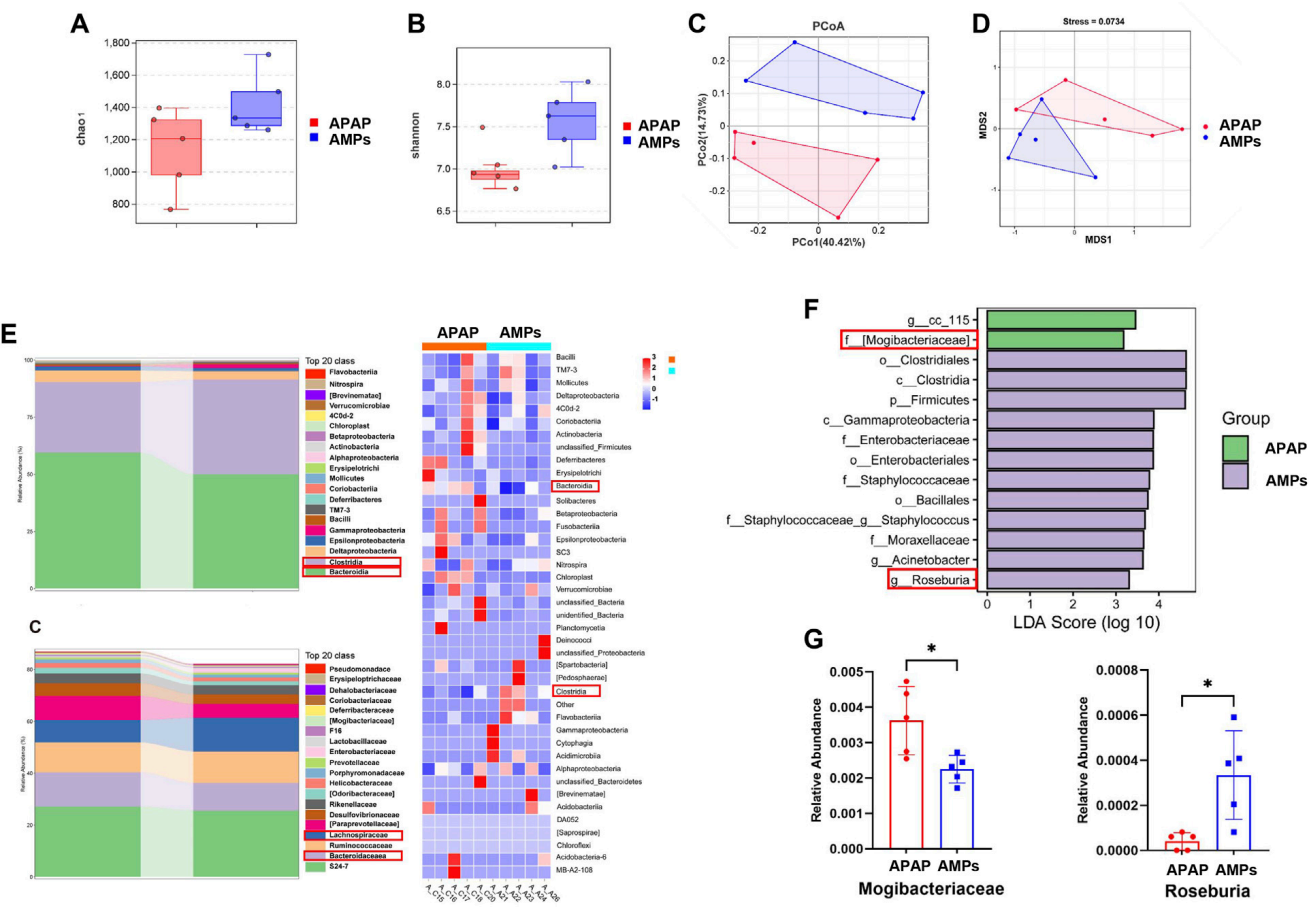
Compositional changes of gut microbiota after AMPs intervention. **(A)** Chao 1 index. **(B)** Shannon index. **(C)** PCoA between groups. **(D)** NMDS between groups. **(E)** Stacked histogram of intestinal flora at the class level and Heat map of intestinal flora at the class level. **(F)** Stacked histogram of intestinal flora at the family level LDA of LEfSe analysis. **(G)** Comparison of the relative abundance of significantly different species in Mogibacteriaceae and Roseburia between groups. Data of biochemical indexes were presented as mean ± SD (n = 5); * indicates APAP Compared with AMPs. *p < 0.05.

To investigate the impact of AMPs on AILI and intestinal microbiota, we analyzed the cecal contents of mice using 16S rDNA sequencing. AMPs significantly increased microbial diversity, with higher α-diversity indices (Chao1 and Shannon) in the AMPs group than in the acetaminophen (APAP) group, suggesting a richer and more balanced microbial community (Figures 5A,B). Multivariate analyses (PCoA and NMDS, Figures 5C,D) showed distinct microbiota profiles between the groups, underlining major compositional differences. β-Diversity metrics demonstrated significant microbial variations between the groups, with notable shifts in bacterial populations at various taxonomic levels. AMPs administration resulted in a decrease in *Bacteroidia* and an increase in *Clostridia* and *Lachnospiraceae* (Figures 5E,F). Lefse analysis identified key differentially abundant taxa, with significant enrichment of *Firmicutes* and reduction in *Mogibacteriaceae* in the AMPs group compared to APAP (Figure 5G). The shifts in microbial communities were linked to therapeutic outcomes, where AMPs elevated beneficial bacteria like *Roseburia*, enhancing gut health and reducing obesity-related inflammation. This family of bacteria has been linked to the promotion of inflammation and apoptosis, with its abundance positively correlating with Bax expression (Lin et al., 2023).

Overall, AMPs significantly improved the intestinal flora in AILI mice, suggesting therapeutic potential through the gut-liver axis by altering critical microbial components. This study underscores the role of AMPs in enriching intestinal biodiversity and shaping a healthier microbial ecosystem, crucial for treating AILI.

### 3.5 Transplant of AMPs-altered gut microbiota protects against APAP-induced liver injury

**FIGURE 6:**
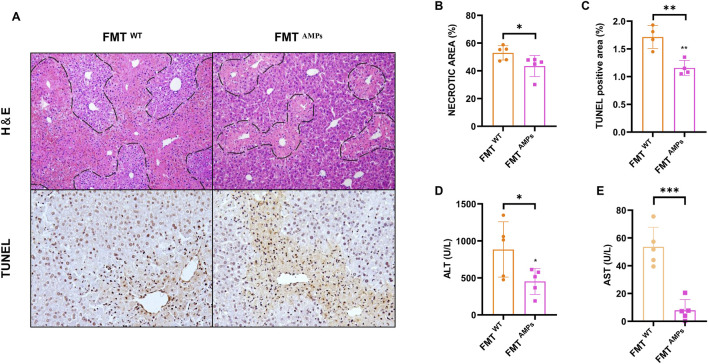
Transplant of AMPs-altered gut microbiota protects against APAP-induced liver injury. **(A)** HE staining and TUNEL immunohistochemical staining of liver. **(B,C)** Statistics of the area of liver pathological necrosis and TUNEL-positive areas. **(D,E)** ALT and AST levels in liver. Data of biochemical indexes were presented as mean ± SD (n = 5); * indicates the comparison between FMT^WT^ group and FMT ^AMPs.^ *p < 0.05, **p < 0.01, ***p < 0.001.

To investigate the role of intestinal flora in AILI, a fecal microbiota transplantation (FMT) experiment was conducted. Liver tissue damage was assessed using H&E staining to examine liver morphology, as shown in Figure 6A. The results revealed that mice receiving AMPs fecal fluid exhibited significantly less damage and bruising around the central vein, along with smaller areas of necrotic cell damage, compared to mice transplanted with wild-type (WT) mouse cecal bacteria. Quantitative analysis of liver injury areas confirmed a significant reduction in the extent of injury in mice treated with AMPs fecal microbiota grafts (*p* < 0.05) (Figure 6B). Additionally, TUNEL staining was performed to evaluate liver cell apoptosis in the mice. The results showed that, compared to the FMT^WT^ group, the number of apoptotic cells near the central vein injury area was significantly reduced in the FMT^AMPs^ group, further demonstrating the protective effect of AMPs in alleviating AILI (Figures 6A,C).

Hepatic ALT and AST levels were measured, with results shown in Figures 6D,E. Mice in the FMT^AMPs^ group showed significantly reduced ALT levels compared to those in the FMT^WT^ group, aligning with the pathological observations. In conclusion, fecal bacterial fluid from AMPs donor mice demonstrated significant therapeutic effects on APAP-induced liver injury. This suggests that the protective effect of AMPs on AILI can be mediated through the intestinal flora, indicating a potential mechanism by which AMPs influences AILI via modulation of the gut microbiome.

### 3.6 Transplant of AMPs-altered gut microbiota into mice ameliorates APAP induced liver inflammation and hepatocyte apoptosis

**FIGURE 7:**
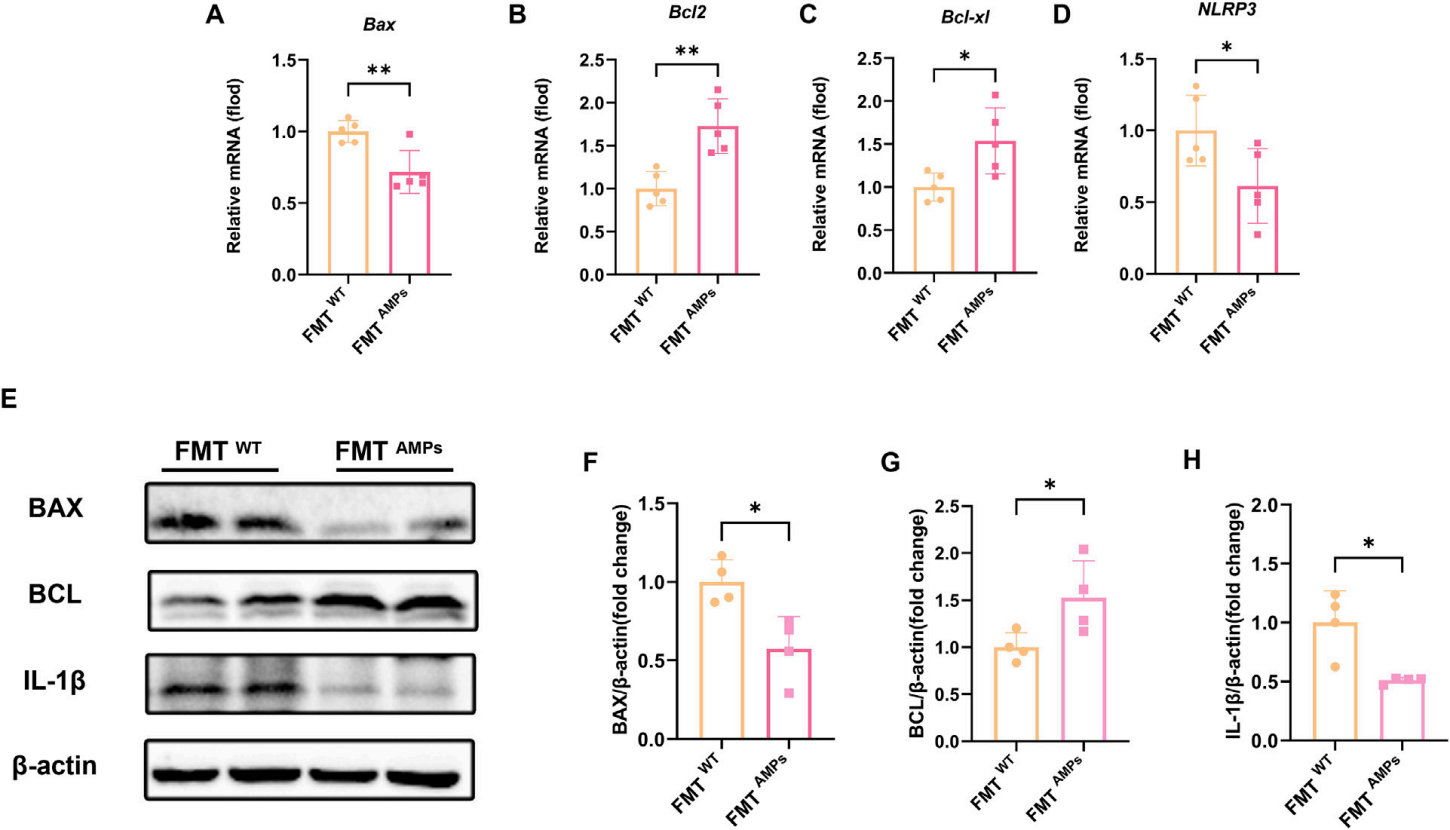
Transplant of AMPs-altered gut microbiota improves liver inflammation and hepatocyte apoptosis in APAP-induced mice. **(A–D)** The *Bax* mRNA, *Bcl-2* mRNA*, Bcl-xl* mRNA and *Nlrp3* mRNA expression level in liver tissue of FMT mice (n = 5). **(E)** Expression levels of related proteins in liver tissue of FMT mice. **(F)** Fold change of BAX protein. **(G)** Fold change of BCL protein. **(H)** Fold change of IL-1β protein, n = 4. * indicates the comparison between FMT^WT^ group and FMT ^AMPs.^ *p < 0.05, **p < 0.01.

To further evaluate the protective effect of fecal microbiota transplantation (FMT) on liver injury, qPCR was conducted to measure the mRNA expression levels of apoptosis markers BAX, BCL-2, BCL-xl, and the inflammatory marker NLRP3 in mice. The results showed that the mRNA levels of *Bax* in the FMT^AMPs^ group were significantly reduced compared to the control group, while the mRNA levels of *Bcl-2* and *Bcl-xl* were significantly increased (Figures 7A–D).

Western blot analysis confirmed these findings. Specifically, the BAX protein level in the FMT AMPs group was significantly lower, while the BCL and IL-1β protein levels were significantly higher (Figures 7E–H). These results are consistent with the gene expression data and further support the idea that AMPs may protect against APAP-induced liver injury by regulating the gut microbiota or by promoting the production of protective metabolites through the microbiota.

This mechanism likely affects liver health through the “gut-liver axis,” where changes in gut microbiota influence liver function. The data suggests that AMPs can reduce APAP-induced apoptosis by modulating the gut microbiota, thereby providing therapeutic benefits in the prevention and treatment of acute liver injury through complex interactions between the gut and liver.

## 4 Discussion

In the development of AILI, the primary hepatotoxic agent causing direct damage to hepatocytes is the drug metabolism by-product N-acetyl-p-benzoquinone imine (NAPQI). When GSH is completely depleted, NAPQI forms covalent bonds in large quantities with mitochondrial membrane proteins. This bonding disrupts the osmotic balance across the mitochondrial membrane, triggering the opening of the mitochondrial permeability transition (MPT) pore. The activation of the MPT pore leads to mitochondrial rupture and a subsequent decrease in ATP production. These process highlights the critical role of mitochondrial integrity in maintaining cellular function and the devastating impact of its disruption during drug-induced liver injury (Nelson, 1990). In acetaminophen-induced liver injury (AILI), a hallmark feature is mitochondrial dysfunction, which triggers DNA damage, apoptosis, and necrosis of hepatocytes, followed by the release of cellular contents. Intracellular components such as nuclear DNA fragments and ATP, acting as damage-associated molecular patterns (DAMPs), are recognized by specific receptors like Toll-like receptors (TLRs) on neighboring cells and immune cells. This recognition drives the release of pro-inflammatory cytokines, including TNF-α and IL-1β (Imaeda et al., 2009). The secretion of these cytokines amplifies the inflammatory response, recruits additional immune cells to the injury site, and exacerbates the inflammatory microenvironment. This cascade not only sustains the inflammatory state but also induces further apoptosis and necrosis of healthy hepatocytes, forming a vicious cycle of liver injury. While this response facilitates tissue regeneration and repair post-AILI, the “second-strike doctrine” also implies an exacerbation of liver damage (Williams, Bajt, Farhood and Jaeschke, 2010).

One prominent feature of APAP-induced liver injury is mitochondrial dysfunction, which compromises cellular respiration and leads to cell asphyxiation. This disruption results in DNA damage, triggering apoptosis and hepatocellular necrosis, and the subsequent release of cellular contents. Intracellular substances, such as nuclear DNA fragments and ATP, are detected by DAMPs receptors, notably the NLRP3 inflammasome, initiating the transcription of pro-inflammatory cytokines (TNF-α, IL-1β, IL-6, and IL-10) (Imaeda et al., 2009). This cascade attracts immune-associated monocytes to the damaged site, eliciting an inflammatory response. While this response facilitates tissue regeneration and repair post-AILI, the “second-strike doctrine” also implies an exacerbation of liver damage. Furthermore, AMPs have demonstrated anti-inflammatory properties across various organs and conditions, supporting its efficacy in liver injury models. Recent studies indicate that apoptosis-related pathways are notably active during AILI, underscoring the significant role of apoptosis in the condition. Following excessive APAP intake, there is a marked increase in the transcription and translation of *B*ax and cysteine proteases, whereas *Bcl-2*, a gene that confers resistance to apoptosis, exhibits significantly reduced expression levels (Miele et al., 2009). Our study has found for the first time that AMPs have a significant therapeutic effect on AILI, manifested in the upregulation of the expression activity of anti-apoptotic genes *Bcl-2* and *Bcl-xl* by AMPs, while downregulating the expression of the pro apoptotic gene Bax, which is a marker of apoptosis, and reducing cell apoptosis; At the same time, AMPs reduce the activation of inflammasome *Nlrp3*, decrease the expression of pro-inflammatory factors such as *Il-1 β*, *Il-6*, and *TNF-α*, and reduce the inflammatory infiltration of macrophages in the damaged area.

The intestinal tract is a critical target organ for metabolic diseases and serves as the habitat for intestinal flora, playing a vital role in various host metabolic processes, including carbohydrate, amino acid, and lipid metabolism, as well as energy and drug metabolism. It is essential for maintaining digestive function, environmental balance, and overall human health (Miele et al., 2009).

The concept of the hepatic-intestinal circulation refers to the bidirectional exchange of substances facilitated by the interconnected biliary and portal systems between the liver and intestines (Morris, 2017). Emerging research has highlighted the critical role of the gut microbiota in influencing AILI. Under normal physiological conditions, intestinal microorganisms contribute to the digestion and metabolism of nutrients while maintaining microbial balance and gut health. However, disruptions to this microbial ecosystem can lead to compositional shifts favoring pathogenic over beneficial bacteria, resulting in reduced microbial diversity. Notable examples of such alterations include the overrepresentation of genera such as *Pseudoflavonifractor* and *Alistipes* (Dey, 2020). This imbalance damages the intestinal mucosal barrier, enabling the translocation of bacteria or harmful metabolites to the liver via the “gut-liver axis,” exacerbating liver damage caused by APAP. Studies have shown that in APAP-induced drug-induced liver injury, the gut microbiota ecosystem is disrupted, leading to significant dysbiosis of bacterial communities such as *Lactobacillus* vaginalis in the intestine. This disturbance reduces the production of bacterial metabolites like β-galactosidase and phenylpropionic acid, thereby exacerbating APAP hepatotoxicity (Cho et al., 2023; Lee et al., 2024). Consequently, in recent years, the intestinal flora has emerged as a pivotal target for managing AILI (Schepers, Glorieux and Vanholder, 2010). To investigate the impact of AMPs on intestinal flora and its therapeutic potential for AILI, we conducted fecal transplantation experiments. These studies confirmed that AMPs influence the intestinal flora, thereby mitigating APAP-induced apoptosis in hepatocytes. This interaction occurs via the hepatic-intestinal circulation, affecting either the flora itself or its metabolites. This suggests that intestinal flora represents a modifiable target for AILI treatment.

Further analysis using 16S rDNA sequencing in our study revealed that AMPs significantly enhances the abundance and balances the composition of intestinal bacteria. This improvement positively affects the health of the intestinal flora. We identified specific bacterial strains responsive to AMPs modulation that contribute to liver injury treatment in AILI. Notably, AMPs increased the abundance of Roseburia, an anti-inflammatory probiotic, and decreased the presence of Mogibacteriaceae, a harmful bacterium linked to increased apoptosis.

## 5 Conclusion

In summary, this study demonstrates that AMPs exert significant protective effects against acetaminophen-induced liver injury by alleviating oxidative stress, suppressing inflammatory responses, and inhibiting hepatocyte apoptosis. In addition, AMPs modulate the gut microbiota composition, thereby contributing to the restoration of the gut-liver axis. Fecal microbiota transplantation experiments further confirmed that the hepatoprotective effects of AMPs are, at least in part, mediated via gut microbial alterations.

Notably, our study provides a comprehensive evaluation of the multi-dimensional therapeutic potential of AMPs in acetaminophen-induced liver injury, incorporating hepatic, immunological, and microbial perspectives. The mechanistic insights into apoptosis regulation, inflammasome suppression, and gut microbiota modulation offer a new angle for considering AMPs as a multi-targeted therapeutic candidate for drug-induced liver injury. These findings merit further investigation and may contribute to the foundation for future translational research and clinical applications in hepatoprotection.

## Data Availability

The data presented in the study are deposited in the NCBI (BioProject) repository, accession number PRJNA1274848.
