# Supplementary material for: Protective effects of Atractylodes macrocephala polysaccharides on acetaminophen-induced liver injury
Source: Front Pharmacol. 2025 Jun 19;16:1583334. doi: 10.3389/fphar.2025.1583334 (PMC12223321; doi:10.3389/fphar.2025.1583334)
Supplement: Supplementary file 1 [file Table1.DOCX]

Supplementary Material

**Supplementary Table 1 Primer sequences for q-PCR**

| **Genes** | **Forward Primers** | **Reverse Primers** |
| --- | --- | --- |
| *Bax* | TGCAGAGGATGATTGCTGAC | GATCAGCTCGGGCACTTTAG |
| *Bcl-2* | GGACTTGAAGTGCCATTGGT | CGGTAGCGACGAGAG AAGTC |
| *Bcl-xl* | TATTGGTGAGTCGGATTGCA | GCTCTCGGGTGCTGTATTGT |
| *Il-1β* | TGGACCTTCCAGGATGAGGACA | GTTCATCTCGGAGCCTGTAGTG |
| *Il-6* | TAGTCCTTCCTACCCCAATTTCC | CTGTTGTTCAGACTCTCTCCCT |
| *Nlrp3* | AGCCTTCCAGGATCCTCTTC | CTTGGGCAGCAGTTTCTTTC |
| *Tnf-α* | CCACCACGCTCTTCTGTCTAC | AGGGTCTGGGCCATAGAACT |
| *β-actin* | GGCTGTATTCCCCTCCATCG | CCAGTTGGTAACAATGCCATGT |
